# Recent Mitochondrial DNA Mutations Increase the Risk of Developing Common Late-Onset Human Diseases

**DOI:** 10.1371/journal.pgen.1004369

**Published:** 2014-05-22

**Authors:** Gavin Hudson, Aurora Gomez-Duran, Ian J. Wilson, Patrick F. Chinnery

**Affiliations:** Wellcome Centre for Mitochondrial Research, Institute of Genetic Medicine, Newcastle University, Central Parkway, Newcastle upon Tyne, United Kingdom; National Institute of Genetics, Japan

## Abstract

Mitochondrial DNA (mtDNA) is highly polymorphic at the population level, and specific mtDNA variants affect mitochondrial function. With emerging evidence that mitochondrial mechanisms are central to common human diseases, it is plausible that mtDNA variants contribute to the “missing heritability” of several complex traits. Given the central role of mtDNA genes in oxidative phosphorylation, the same genetic variants would be expected to alter the risk of developing several different disorders, but this has not been shown to date. Here we studied 38,638 individuals with 11 major diseases, and 17,483 healthy controls. Imputing missing variants from 7,729 complete mitochondrial genomes, we captured 40.41% of European mtDNA variation. We show that mtDNA variants modifying the risk of developing one disease also modify the risk of developing other diseases, thus providing independent replication of a disease association in different case and control cohorts. High-risk alleles were more common than protective alleles, indicating that mtDNA is not at equilibrium in the human population, and that recent mutations interact with nuclear loci to modify the risk of developing multiple common diseases.

## Author Summary

There is a growing body of evidence indicating that mitochondrial dysfunction, a result of genetic variation in the mitochondrial genome, is a critical component in the aetiology of a number of complex traits. Here, we take advantage of recent technical and methodological advances to examine the role of common mitochondrial DNA variants in several complex diseases. By examining over 50,000 individuals, from 11 different diseases we show that mitochondrial DNA variants can both increase or decrease an individual's risk of disease, replicating and expanding upon several previously reported studies. Moreover, by analysing several large disease groups in tandem, we are able to show a commonality of association, with the same mitochondrial DNA variants associated with several distinct disease phenotypes. These shared genetic associations implicate a shared underlying functional effect, likely changing cellular energy, which manifests as distinct phenotypes. Our study confirms the important role that mitochondrial DNA variation plays on complex traits and additionally supports the utility of a GWAS-based approach for analysing mitochondrial genetics.

## Introduction

Mitochondria are the principal source of cellular adenosine triphosphate (ATP) generated through oxidative phosphorylation (OXPHOS), which is linked to the respiratory chain. In humans, thirteen OXPHOS proteins are synthesised from the 16.5 Kb mitochondrial genome (mtDNA). MtDNA has accumulated genetic variants over time, and being strictly maternally inherited, undergoes negligible intermolecular recombination. As a consequence, ancient variants extant in the human population define haplogroups that have remained geographically or ethnically restricted [1]. Work on European haplogroups has shown that some polymorphic mtDNA variants affect mitochondrial function [2], [3].

Given emerging evidence that mitochondria play a key role in several common diseases, it is likely that variation of mtDNA could alter the risk of developing different human disorders. Early mtDNA genetic association studies were under-powered, and the vast majority have not been replicated [4]. However, some recent large studies have found replicable associations with specific human diseases [5]–[11], most notably in sporadic Parkinson's disease [12]–[14]. These observations implicate mtDNA as part of the “missing heritability” of complex human disease traits.

Ultimately, mtDNA codes for a limited number of proteins that affect the same common pathway of energy production implicated in several human diseases. It is likely, therefore, that functional genetic variation of mtDNA will have impact on more than one disease – but this has not been directly studied before. To test this hypothesis, we analysed mtDNA SNP data from 51,106 subjects from the Wellcome Trust Case Control Consortium, comparing genotypes from 11 major diseases: ankylosing spondylitis (AS, n = 2,005), ischemic stroke (IS, n = 4,205), multiple sclerosis (MS, n = 11,377), Parkinson's disease (PD, n = 2,197), primary biliary cirrhosis (PBC, n = 1,921), psoriasis (PS, n = 2,622), schizophrenia (SP, n = 2,019), ulcerative colitis (UC, n = 2,869), coronary artery disease (CAD, n = 3,215), hypertension (HT, n = 2,943) and type-2 diabetes (T2D, n = 2,975) to three independent control groups genotyped on the same platforms (WTCCC-58C, n = 2997, WTCCC-NBS, n = 2897 and WTCCC2-MetabaloChip, n = 5841).

## Results

### Common mtDNA variants are associated with common disease

After applying stringent quality control measures (**Supplementary Materials, Table S1 & S2**), we initially compared the two healthy control groups using PLINK v2.050 [15] (**Supplementary Materials, Figure S1**), and found no significant difference in allele frequencies. We therefore merged control groups genotyped on the same platform for all subsequent analyses as follows: WTCCC-Control-1, WTCCC-Control-2 and WTCCC-Control-3 (**Supplementary Materials, Table S2**).

Cluster plots produced by principle component analysis (PCA) revealed no significant population stratification when comparing either: datasets from the same array or array-specific control datasets (**Supplementary Materials, Figure S4**).

**Figure 1:**
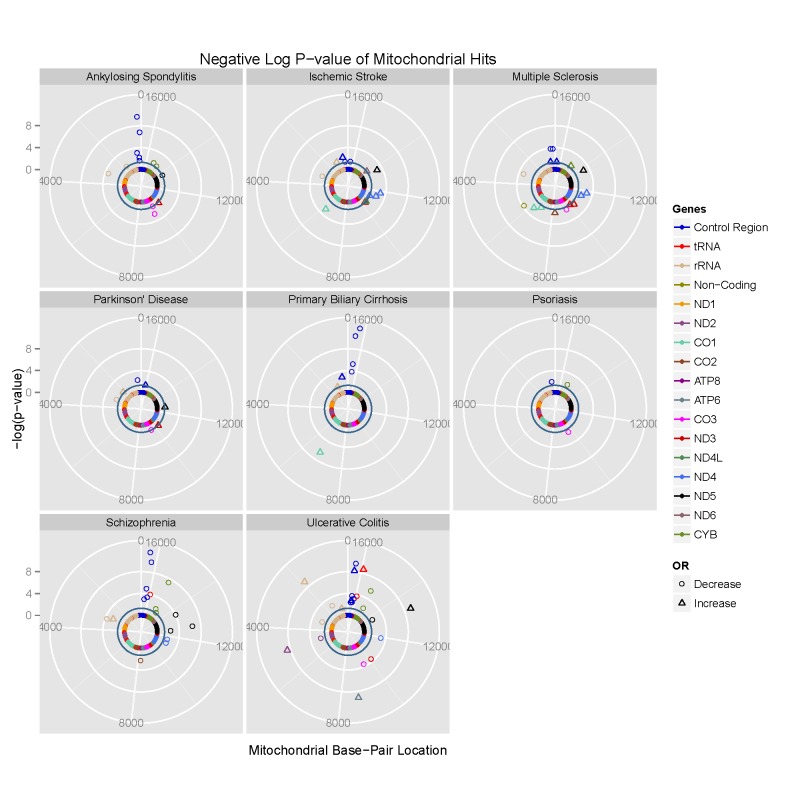
Circularised Manhattan plots of imputed P values showing the association between mtDNA variants and eight complex traits. Radial axis: –log(P-value) (where dark inner circle indicates P = 0.05 threshold). Circumference: position on the mitochondrial genome based on the revised Cambridge Reference Sequence (rCRS, NC_012920) numbering in an anti-clockwise direction from 12 o'clock. Each gene is colour coded, as shown on the figure.

We then compared genotyped SNPs in each disease group to platform-matched control datasets using PLINK v2.050 [15] (**Figure 1** **& Supplementary Materials, Table S3**). This confirmed previously reported associations at the low-resolution haplogroup level [5], [12], [16], [17], endorsing the methodological approach.

### Phylogenetically-related mtDNA variants are associated with common disease

Next we performed lexical tree building to identify new associations with phylogenetically related variants, but without basing our anlysis on any prior assumptions related to the published mtDNA haplogroup structure [18], [19]. This method uses fewer SNPs because individuals with missing SNP data cannot be used, but has greated power, and provides graphical summaries of the combinations of SNPs that are associated with increased or descreased risk of disease (**Supplementary Materials, Table S4**). Lexical tree analysis identified significant relationships between the mtDNA tree structure and schizophrenia, primary biliary cirrhosis, multiple sclerosis (each at p<10^−6^), ulcerative colitis (p<10^−4^), and Parkinson's disease (p = 0.004) (**Table 1** **and Supplementary Materials Figure S3**), independently confirming previous haplogroup based association associations [5], [12], [16], [17], and revealing new mtDNA clades associated with several different diseases. The other case-control trees, and comparisons between the different control populations were not significant at the 1% level.

**Table 1:** Lexical tree analysis.

Disease	Array	Removed by QC	Rare haplotypes (<5 or not in controls) removed	Sample Size	Whole Tree p-value
Ankylosing Spondylitis	Illumina 610K	243	11	1751	0.30
Ischemic Stroke	Illumina 610K	407	41	3757	0.015
Multiple Sclerosis	Illumina 610K	1317	74	9985	<1E-6
Parkinson's Disease	Illumina 610K	235	6	1956	0.19
Primary Biliary Cirrhosis	Illumina 610K	145	11	1765	<1E-6
Psoriasis	Illumina 610K	285	7	2330	0.004
Control NBS	Illumina 610K	127	4	2597	-
Control 58C	Illumina 610K	171	7	2752	-
Type-2 Diabetes	MetabaloChip	117	1	2857	0.085
Coronary Artery Disease	MetabaloChip	46	0	3079	0.30
Hypertension	MetabaloChip	87	0	2856	0.12
Control	MetabaloChip	150	0	5691	-
Ulcerative Colitis	Affymetrix SNP 6.0	172	7	2690	1.5E-5
Schizophrenia	Affymetrix SNP 6.0	115	5	2950	<1E-6
Control NBS	Affymetrix SNP 6.0	149	12	2826	-
Control 58C	Affymetrix SNP 6.0	190	11	2796	-

Quality control (QC), sample sizes and tests for association between disease and the tree. P values were estimated using 10^6^ permutations of the tree labels.

### Imputed mtDNA variants are associated with several different common diseases

To determine the functional basis of the associations we imputed missing genotypes across the whole mitochondrial genome using 7,729 complete mtDNA sequences. Subsequent analyses were performed on 35,901 European cases and 15,302 European controls, and captured 40.41% of European mtDNA population genetic variation (**Supplementary Materials, Figure S2**).

In keeping with our original hypothesis, specific variants with predicted functional consequences conferred either an increased risk (**Table 2a**) or decreased risk (**Table 2b**) across several different diseases. In addtion, we identified the same allelic-specific associations for different diseases compared to different platform-specific control groups, re-inforcing these findings. Functional variants associated with an increased risk in two or more diseases were limited to two structural genes: *MTCYB* (m.14793, m.15218) and *MTCO3* (m.9477, m.9667). The only non-synonmous protien encoding variant consistently associated with a reduced risk of disease was in *MTND3* (m.10398).

**Table 2:** Imputed mitochondrial DNA variants associated with more than one complex disease at p<0.05.

*a*)	*Variant*		***m.310***	***m.3197***	*m.9477*	*m.9667*	***m.13617***	*m.14793*	***m.15043***	*m.15218*
	*mtDNA haplogroup*		***U4a2***	***U5***	*U5*	*J1b2a/U5a1b*	***U5***	*U5a*	***N1a1***	*U5a1*
	*Gene/region*		***D-loop***	***16S rRNA***	*MT-CO3*	*MT-CO3*	***MT-ND5***	*MT-CYB*	***MT-CYB***	*MT-CYB*
	*AA substitution*	*Control Used*	*-*	*-*	*V91I*	*N154S*	***Syn***	*H16R*	***Syn***	*T158A*
	*Schizophrenia*	*WTCCC-Control 2*	*-*	*2.3 (1.1×10^-4^)*	*-*	*-*	*1.51 (8.6×10^-5^)*	***1.28*** * (3.8×10^-2^)*	*1.41 (1.1×10^-2^)*	***2.87*** * (5.0×10^-8^)*
	*Ulcerative Colitis*	*WTCCC-Control 2*	*-*	*1.33 (9.2×10^-2^)*	***1.48*** * (2.5×10^-2^)*	*-*	*1.32 (1.1×10^-2^)*	*-*	*1.44 (7.6×10^-2^)*	***2.60*** * (2.9×10^-6^)*
	*Ankylosing Spondylitis*	*WTCCC-Control 1*	*1.30 (8.9×10^-4^)*	*1.53 (4.4×10^-4^)*	***1.45*** * (1.9×10^-3^)*	***1.73*** * (4.6×10^-2^)*	*1.26 (4.5×10^-2^)*	***1.34*** * (2.8×10^-2^)*	*-*	***1.48*** * (1.6×10^-2^)*
	*Multiple Sclerosis*	*WTCCC-Control 1*	*1.20 (1.6×10^-4^)*	*1.32 (7.7×10^-4^)*	***1.22*** * (1.4×10^-2^)*	*-*	*-*	*-*	*-*	*-*
	*Ischemic Stroke*	*WTCCC-Control 1*	*1.13 (3.9×10^-2^)*	*1.30 (1.0×10^-2^)*	*-*	*-*	*-*	*-*	*-*	*-*
	*Parkinson's' Disease*	*WTCCC-Control 1*	*1.25 (5.4×10^-3^)*	*1.36 (1.7×10^-2^)*	*-*	*-*	*-*	*-*	*-*	*-*
	*Psoriasis*	*WTCCC-Control 1*	*1.71 (1.1×10^-2^)*	*-*	*-*	***1.88*** * (1.2×10^-2^)*	*-*	*-*	*-*	*-*

*b)*	*Variant*		***m.497***	***m.1189***	***m.6260***	***m.6776***	*m.10398*	***m.11485***	***m.11840***	***m.13740***
	*mtDNA haplogroup*		***K***	***K***	***Homoplastic(H/K)***	***H***	*Homoplastic(J/K)*	***K***	***K***	***Homoplastic(H/K)***
	*Gene/region*		***D-loop***	***12S rRNA***	***MT-CO1***	***MT-CO1***	*MT-ND3*	***MT-ND4***	***MT-ND4***	***MT-ND5***
	*AA substitution*		*-*	*-*	***Syn***	***Syn***	*T114A*	***Syn***	***Syn***	***Syn***
	*Multiple Sclerosis*	*WTCCC-Control 1*	0.82 *(3.6×10^-2^)*	-	0.63 *(1.4×10^−2^)*	0.78 *(2.1×10^-2^)*	**0.87** *(1.5×10^-2^)*	0.67 *(8.3×10^-3^)*	0.58 *(1.2×10^-3^)*	0.58 *(1.3×10^-3^)*
	*Ischemic Stroke*	*WTCCC-Control 1*	0.72 *(5.0×10^-3^)*	0.76 *(1.6×10^-2^)*	0.53 *(1.3×10^−3^)*	-	**0.88** *(4.9×10^-2^)*	0.57 *(3.6×10^-3^)*	0.49 *(8.8×10^-4^)*	0.49 *(9.5×10^-4^)*
	*Primary Biliary Cirrhosis*	*WTCCC-Control 1*	0.58 *(1.3×10^-2^)*	0.74 *(3.8×10^-2^)*	-	0.30 *(3.7×10^-7^*)	**-**	-	-	-
	*Ankylosing Spondylitis*	*WTCCC-Control 1*	-	-	-	-	**0.83** *(3.7×10^-2^)*	-	-	-
	*Parkinson's Disease*	*WTCCC-Control 1*	-	-	-	-	**0.84** *(4.4×10^-2^)*	-	-	-

*c)*	*Variant*		***m.11299***	***m.16294***						
	*mtDNA haplogroup*		***K***	***Homoplastic***						
	*Gene/region*		***MT-ND4***	***D-loop***						
	*AA substitution*		***Syn***	*-*						
	*Ischemic Stroke*	*WTCCC-Control 1*	0.81 *(4.6×10^−2^)*	-						
	*Primary Biliary Cirrhosis*	*WTCCC-Control 1*	-	1.80 *(1.6×10^−4^)*						
	*Schizophrenia*	*WTCCC-Control 2*	1.29 *(9.6×10^−3^)*	-						
	*Ulcerative Colitis*	*WTCCC-Control 2*	-	0.43 *(6.1×10^−9^)*						

Odds ratios (and P value) showing: (a) increased risk; (b) decreased risk; (c) and opposite risks. Variant position is based on the revised Cambridge Reference Sequence for mtDNA (rCRS, NC_012920). AA = amino acid.

**Figure 2:**
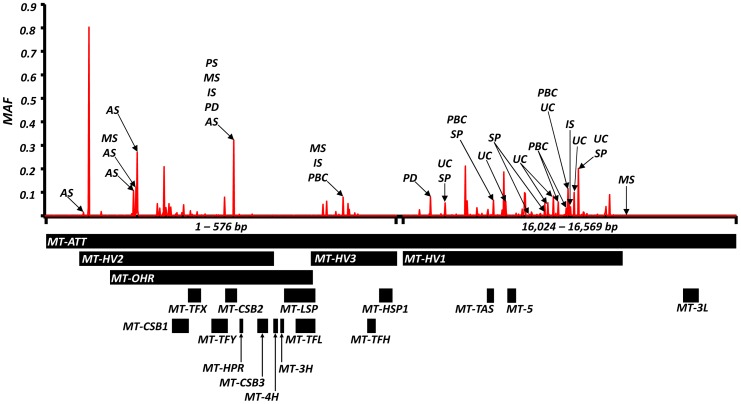
High-resolution map of the non-coding region of mtDNA (d-loop) showing allele frequencies in 7,729 control subjects and the position of alleles associated with eight common human diseases; where: AS = ankylosing spondylitis, IS = ischaemic stroke, MS = multiple sclerosis, PD = Parkinson's disease, PBC = primary biliary cirrhosis, UC = ulcerative colitis and SP = schizophrenia. Alleles associated with multiple diseases are shown in Table 2. Alleles associated with single diseases are shown in Table S1. Y-axis: minor allele frequency (MAF) in 7,729 control subjects. X-axis: position on the mitochondrial genome based on the revised Cambridge Reference Sequence (rCRS, NC_012920) with corresponding regions annotated below.

We also found evidence of associations across multiple diseases within the non-coding region (d-loop) of mtDNA, and 16S ribosomal RNA subunit genes (**Figure 2** and **Table 2** and **Supplementary Materials, Table. S3**). Intriguingly, the same alleles were not associated with all of the diseases we studied, and for two variants (m.11299, m.16294), the same allele had opposite effects for two different diseases (**Table 2c**).

Overall, the majority of disease-associated alleles conferred an increased risk (61/99), and not a decreased risk (38/99, P<0.001) (**Supplementary Materials**, **Table S3**).

## Discussion

Following stringent quality control, our initial analysis confirmed previous associations between mtDNA haplogroups and common disease in a much larger data set. These findings were independentely supported by lexical tree based analysis at higher levels of statistical significance. Subsequent imputation of missing genotypes captured >40% of European mtDNA population genetic variation in 35,901 European cases and 15,302 European controls. By simultaneously analysing eleven, ostensibly unrelated, diseases we identified several imputed mtDNA variants that were associated with more than one disease. The same associations were seen in different disease groups compared to different control groups. This provided confirmatory independent replication of a disease association, and supports our original hypothesis that the same genetic variants of mtDNA contribute to the risk of developing several common complex diseases.

Variants increasing the risk of two or more diseases were limited to *MTCYB* (m.14793, m.15218) and *MTCO3* (m.9477, m.9667), encoding variants in cytochrome *b* (H16R, T158A) and subunit 3 of cytochrome *c* oxidase (complex IV, V91L, N154S). Functional variants of *MTCYB* have previosly been associated with several human phenotypes [20]–[22], but the most compelling evidence of a prior disease association is the increased risk of developing blindness in subjects harboring the mtDNA mutations in *MTND* genes known to cause Leber hereditary optic neuropathy (LHON), where they synergistically interact with a primary LHON mutation to cause a defect of OXPHOS complex I activity [23]. On the other hand, the only non-synonmous protien encoding variant associated with a reduced risk of several diseases was m.10398 in the *MTND3* variant (complex I, T114A). m.10398 occurs twice on the human mtDNA phylogeny (homoplastic on haplogroups J and K), and has previously been associated with a reduced risk of Parkinson's disease [14], [24]. This variant has been shown to reduce complex I activity, cytosolic calcium levels, and the mitochondrial membrane potential [3], [25], [26] and thus may reduce the level of reactive oxygen species, contributing to the underlying disease mechanim of several disorders.Variants in *MTCO3* are typically associated with primary mitochondrial disorders [27], [28], but have been also been indentified as risk factors in Alzheimer's disease [29], [30], migrainous stroke [31] and sporadic optic neuropathy [32]. M.9477 and m.9667 are non-synonmous protien encoding variants which are cladally related; present on haplogroup U sub branches (U5 and U5a1b, respectively). Cybrid studies of haplogroup U show a reduction in mtDNA copy number, resulting in a reduction in mitochondrial protein synthesis and complex IV activity [3], [25], impairing energy production and likely contributing to disease.

We also noted disease associations with substitutions in the non-coding region and ribosomal genes (**Table 2** and **Supplementary Materials, Table S3**). Although highly polymorphic at the population level (**Figure 2**), there is emerging evidence that both regions can have functional effects either through an effect on mtDNA replication, transcription or translation [33], [34], as proposed in Alzheimer's disease [34].

It is intriguing that there were more functional variants associated with an increased risk, than with a decreased risk of disease (**Table 2** and **Supplementary Materials, Table S3**). This suggests that deleterious, novel sub-haplogroup variants have not yet been removed from the population through natural selection, possibly including the younger d-loop variants. This has been observed in the nuclear genome in the rapidly expanding human population [35], [36], implying that the modern human population is far from equilibrium. An alternative explantion is that mtDNA alleles may escape purifying selection because the associated disease phenotype only becomes manifest after female reproductive life.

For two variants (m.11299, m.16294), the same allele was associated with an increased risk of developing one disease, and a reduced risk of developing another (**Table 2**). Although differences in the sample size post-QC provide one explanation, these findings raise the possibility that different mtDNA-mediated mechanisms are involved in different contexts, perhaps because some variants have a greater impact on bioenergetics, and others on the generation of reactive oxygen species. Alternatively, it is conceivable that the relevance of specific alleles may be context-specific, only excerting a functional effect on a particular haplogroup background [37]. Substantially larger whole mtDNA genome studies will be required to detect clade-specific epistastic interactions if they exist.

In some instances we observed multiple associations with different variants found within the same phylogenetic cluster. For example m.499 (K1a), m.11485 (K1a4) and m.11840 (K1a4a1) are known to reside within subdivisions of the major haplogroup K, and all associated decreased risk of MS and IS. Conversely, m.310 (U4a2) and m.3197 (U5) are distinct subclades of the U associated with increased risk of PS, MS, IS PD AS and UC. Although reassuring from a technical perpective, this illustrates the challenge of mtDNA association studies, where variants with a close ancestral relationship inevitably co-segregate, making it difficult to determine which alleles are responsible for the disease risk.

Finally, analysis of imputed data also revealed several different mtDNA alleles asssociated with different diseases, often reaching high levels of statistical significance (P<10^−10^, **Supplementary Materials, Table S3**). However, these findings should only be considered preliminary and require independent replication in other populations (where specific European haplogroup distributions can vary) and thus do not form the major focus of this report.

In conclusion, these findings underscore the role of mitochondrial mechanisms in the pathogenesis of common diseases, and emphasise the importance of incorporating the mitochondrial genome in comprehensive genetic association studies. Although the strict phylogenetic stucture of maternally inherited mtDNA makes it difficult to identify the precise variants responsible, higher resolution genotyping at the whole mtDNA genome level will cast further light on the genetic mechanisms, particularly if recurrent homoplasies independently associate with phenotypes across several clades.

## Materials and Methods

This study used data generated through the Welcome Trust Case Control Consortium. A full list of the corresponding investigators who generated each dataset is available from http://www.wtccc.org.uk/ccc2/wtccc2_studies.html [38]–[45]. Both case and control datasets were downloaded from the European Genotype Archive (http://www.ebi.ac.uk/ega).

Psoriasis (PS), multiple sclerosis (MS), ischemic stroke (IS), Parkinson's disease (PD), primary biliary sclerosis (PBC) and ankylosing spondylitis (AS) patient cohorts were genotyped using the Illumina 610K quad array (Illumina San Diego California USA) and were compared array specific controls, denoted here as WTCCC-Control-1 (combined WTCCC-58C and WTCCC-NBS) genotyped on the Illumina 1.2M Duo platform (Illumina San Diego California USA). Illumina array systems contain 138 mtDNA variants.

Ulcerative colitis (UC), schizophrenia (SP) and their array-specific controls, denoted here as WTCCC-Control-2 (combined, WTCCC-58C and WTCCC-NBS), were genotyped using the Affymetrix SNP6.0 array (Affymetrix, Santa Clara, CA). The Affymetrix SNP6.0 array system contains 445 mtDNA variants.

Coronary artery disease (CAD), Type-2 diabetes (T2D) and hypertension (HT) cohorts and their array specific controls, denoted here as WTCCC-Control-3 (combined WTCCC-58C and WTCCC-NBS), were genotyped using the MetabaloChip array system [46]. The MetabaloChip array system contains 135 mtDNA variants.

To ensure valid comparisons, each disease sample set wasonlycompared to its corresponding control array counterpart(i.e. SNP6.0 cases were compared to SNP6.0 controls).”

### Statistical power

Given the case cohort sample sizes post QC (Supplementary Materials, Table S1), the corresponding control cohorts (Supplementary Materials, Table S1), *an expected MAF of 0.01, an α = 3.85×10^−3^ to 3.97×10^−4^(averaging 13-126 tests dependent upon specific dataset*) and disease prevalences of: psoriasis = 2% [47], multiple sclerosis = 1% [48], ischemic stroke = 1% [49], primary biliary cirrhosis = 0.1% [50], Parkinson's disease = 0.3% [51], ankylosing spondylitis = 0.1% [52], ulcerative colitis = 0.1% [53], schizophrenia = 0.33% [54], Type-2 diabetes = 10% [55], coronary artery disease = 3% [56] and hypertension = 30% [57]; we had >80% power to detect an effect size of >1.2 in each cohort (specifically, psoriasis = 79.8%, multiple sclerosis = 93.2%, ischemic stroke = 84.5%, primary biliary cirrhosis = 79.9%, Parkinson's disease = 85.9%, ankylosing spondylitis = 85.4%, ulcerative colitis = 78.9%, schizophrenia = 80.3%, Type-2 diabetes = 85.3%, coronary artery disease = 82.6% and hypertension = 98.7%). Power calculations were carried out using Genetic Power Calculator [58].

### Primary association analysis

Stringent quality control (QC) was applied to each individual cohort (**Table S1**) [59]. Briefly, each cohort was pruned of missing phenotypes (defined as -9 in the pedigree/sample files). Poorly performing SNPs (genotyped = 0.1[59]), and subsequenctly, samples were removed (individual missingness  = 0.1 [59]) using PLINK v2.050 [15]. Additionally non-European mtDNA sequences (defined with m.8701A, m.8540T and 10873T) were also removed [1], [60], [61]. Finally, to verify the quality of genotypes cluster plots of normalized intensity for each SNP were generated using R (http://www.R-project.org) and inspected.

In order to increase statistical power, WTCCC-58C and WTCCC-NBS control cohorts were merged. Initially, we compared the two healthy control groups (**Supplementary Materials, Figure S1**), and found no significant difference in allele frequencies. Briefly, each control cohort was merged with its array genotyped counterpart (**Supplementary Materials, Table S2**). As with individual cohorts, MAF = 0.00001, implemented in PLINK v2.050 [15], was used to remove SNPs with missing genotpyes (i.e. call = 0 0). Poorly performing SNPs (genotyped = 0.1[59]), and subsequenctly, samples were removed (individual missingness = 0.1[59]) using PLINK v2.050 [15]. Finally, to correct for control popualtion stratitification, variant frequency was compared between -58C and –NBS using ‘—assoc' PLINK v2.050 (P threshold = 0.05) [15]. Variants with signifcantly different 58C/NBS frequencies were removed. This QC lead to the formation of 3 merged control cohorts: WTCCC-Control-1, WTCCC-Control-2 and WTCCC-Control-3.

Prior to association testing QC'd case cohorts were merged with corresponding QC'd control cohorts (i.e. Multiple sclerosis versus WTCCC-Control-1). Differential missingness tests, which statistically compare the frequency of ‘missing’ genotype data between cases and controls were performed on each case-control comparison [59]. Variants were removed when missingness was significantly different (P = <10^−4^) [59]. Allelic association was implemented in PLINK v2.050 [15]. Given the discovery nature of the experiment, statistical significance was defined as P<0.05.

### Population stratification

Only ancestral Europeans, determined by mitchondrial DNA genotype, were included in this study [1], [60], [61]. Additionally, population structure in each cohort (post-QC) and combined by array type was assessed by principle component analysis (PCA) of mitochondrial DNA variants [62]. Plots were made of the first two components for each array dataset (Illumina = AS, IS, MS, NBS, PBC, PD, PS, WTCCC-58C and WTCCC-NBS, Affymetrix = SP, UC, WTCCC-58C and WTCCC-NBS and Metabalo = T2D, CAD, HT and controls [previously combined WTCCC-58C and WTCCC-NBS]) and separately for the control cohorts for each platform (Supplementary Figure S3). At this resolution, individual PCA cluster analysis showed no significant stratification differences. All principle component scores were calculated in R using the ‘princomp’ function and plotted in R using ggplot (R Core Team 2013) [63].

### Imputation

Imputation was implemented in PLINK v2.050 [15]. Initially a reference panel was constructed. Whole Human mtDNA genome data, n = 18,114 sequences, were downloaded from the National Centre for Biotechnology Information Nucleotide database (http://www.ncbi.nlm.nih.gov/nuccore/), using the keyword phrase ‘Homo [Organism] AND gene_in_mitochondrion[PROP] AND 14000∶19000[SLEN] NOT pseudogene[All Fields]'. Sequences with pathogenic mtDNA variants (available at www.mitomap.org) were removed (n = 458 sequences), non *Homo sapien* sequences were removed (n = 7). Similar to genotype QC, non-European mtDNA sequences (defined with m.8701A, m.8540T and 10873T) were also removed (n = 7051). Finally truncated mtDNA sequences (<16,500 bp) were removed (n = 663) leaving a final dataset of n = 9,935 sequences. The sequence dataset was aligned using MUSCLE [64], analysed using Haplogrep [65], [66] and subsequently filtered to match the Major European haplogroups (H, V, J, T, U, K, W, X, I, R and N) leaving a final sequence aplosamples and 2,873 variants, representing 100% of of the genetic varation in the reference dataset.

The reference panel was merged with each QC'd case-control cohort in PLINK (v2.050),[15] invoking ‘—flip-scan' to detect and correct any stranding issues. Imputation association testing was carried out using ‘—proxy-assoc’ and, in order to assess the imputation performance, ‘—proxy-drop’.[15] Significant SNPs associations with >99% of samples imputed, number of proxy SNPS >3, a MAF >0.01 and a content metric >0.8 were retained.[15] Given a popualtion size of 7,729 and total genotypic information of 2,873 as 100%, imputation of alleles with MAF>0.0 captures 40% of total mtDNA genetic variabilty (**Figure S2**).

### Circularised Manhattan plot

Cicularised Manhattan plots were generated using code adapted from http://gettinggeneticsdone.blogspot.co.uk/2013/11/a-mitochondrial-manhattan-plot.html, solarplot.R and ggplot2 (http://ggplot2.org/).

### Lexical tree analysis

Lexical tree analysis was performed in R (R Core Team 2013) [63] using a custom library (snptree, publically available from http://www.staff.ncl.ac.uk/i.j.wilson/). This analysis was performed on the Illumina 610K quad array, the Affymetrix SNP6.0 and the MetabaloChip datasets independently. An independent stringent QC was performed, removing in order: the SNPs with a call rate of below 95% or a MAF of below 0.5%, the 2% of individuals with the most missing sites, the bottom 50% of SNPs with the most missing samples at that site, and those individuals with any missing data from the remaining SNPs. Finally, those individuals with haplotypes (defined by all the remaining SNPs) that were not present in controls or had a frequency of less than 5 were removed. This left 27054 individuals on 24 SNPs for the Illumina 610K quad array, 10,745 individual at 15 SNPs for the Affymetrix 6.0 chip and 14,484 individuals at 5 SNPs for the MetabaloChip. The SNPs retained and their minor allele frequencies (MAF) in the control populations are shown in **Supplementary Materials, Table S4**. A tree structure was contructed for haplotypes made from the retained SNPs by initially grouping all individuals at the root of a tree, and then successively considering all retained SNPs in decreasing order of their minor allele frequency (**Supplementary Materials, Figure S3**). At each stage, the haplotypes at each leaf node are split with those with the wild type being put on the left branch and those with the mutant allele on the right. This creates a tree with all leaves representing complete haplotypes and internal nodes partial haplotypes. Test statistics were then calculated for each node on the tree. An overall test statistic for the tree was calculated by calculating the the sum of the five largest node values that were not ancestors or descendents of each other. The test statistic was tested for significance by 1,000,000 random permutations of the Case/Control labels.

## Supporting Information

Figure S1 Mitochondrial DNA control allele frequencies after quality control (see methods). Comparison of the WTCCC-58C and WTCCC-NBS control cohorts. Solid line  =  linear regression for the Illumina data. Dotted line  =  linear regression for the Affymetrix data. MAF = minor allele frequency.
(DOCX)

Figure S2 Frequency distribution histogram showing the percentage frequency of mitochondrial DNA variants in the imputation reference panel (2,873 variants from 7,729 subjects) plotted against the minor allele frequency (MAF) in the reference panel. 40.41% variants have a MAF>0.01. These variants were included in the imputation analysis.
(DOCX)

Figure S3 Lexical tree analysis of complex trait SNP data. Shown are skeletal tree configurations for each genotyping platform, control data comparisons (WTCCC-58C versus WTCCC-NBS for each platform, showing no significant haplotype associations) and case-control comparisons on each skeletal tree. Numbers indicate nodal frequencies and significant associations are highlighted in colour, where blue boxes indicate a protective haplotype association. AS = Ankylosing spondylitis, IS = ischaemic stroke, MS = multiple sclerosis, PD = Parkinson's disease, PBC = primary biliary cirrhosis, PS = psoriasis, SP = schizophrenia, UC = ulcerative colitis, CAD = coronary artery disease, HT = hypertension, and T2D = type 2 diabetes.
(DOCX)

Figure S4 **–** PCA analysis of mtDNA variants, showing clustering of: *a*) combined Illumina array cohorts (AS, IS, MS, NBS, PBC, PD, PS, WTCCC-58C and WTCCC-NBS); *b*) Illumina genotype controls only (WTCCC-58C and WTCCC-NBS); *c*) combined Affymetrix array cohorts (SP, UC, WTCCC-58C and WTCCC-NBS); *d*) Affymetrix genotype controls only (WTCCC-58C and WTCCC-NBS) and *e*) combined Metabalo array cohorts (T2D, CAD, HT and controls [previously combined WTCCC-58C and WTCCC-NBS]).
(DOCX)

Table S1 Impact of quality control procedure on the number of samples and genotypes (see methods). MAF = minor allele frequency.
(DOCX)

Table S2 Impact of quality control procedure when combining control data (see methods).
(DOCX)

Table S3 Association between imputed mitochondrial DNA variants and eight complex diseases, showing the corresponding control cohort, array SNP ID, variant position in the mitochondrial genome (rCRS, NC_012920), minor allele frequency in cases and controls (A1-cases and A1-Cont. respectively), case-control comparison (chi-square test P, *na* = not available in primary analysis), imputed significance (P) and odds ratio (OR). Hap = corresponding major and sub mitochondrial haplogroup.
(DOCX)

Table S4 Mitochondrial DNA variants used to the lexical tree analysis (see methods). Variant position in the mitochondrial genome is based on the revised Cambridge reference sequence (rCRS, NC_012920).
(DOCX)
