# Supplementary figures and images for: Recent Mitochondrial DNA Mutations Increase the Risk of Developing Common Late-Onset Human Diseases
Source: PLoS Genet. 2014 May 22;10(5):e1004369. doi: 10.1371/journal.pgen.1004369 (PMC4031051; doi:10.1371/journal.pgen.1004369)

***Figure S1.***


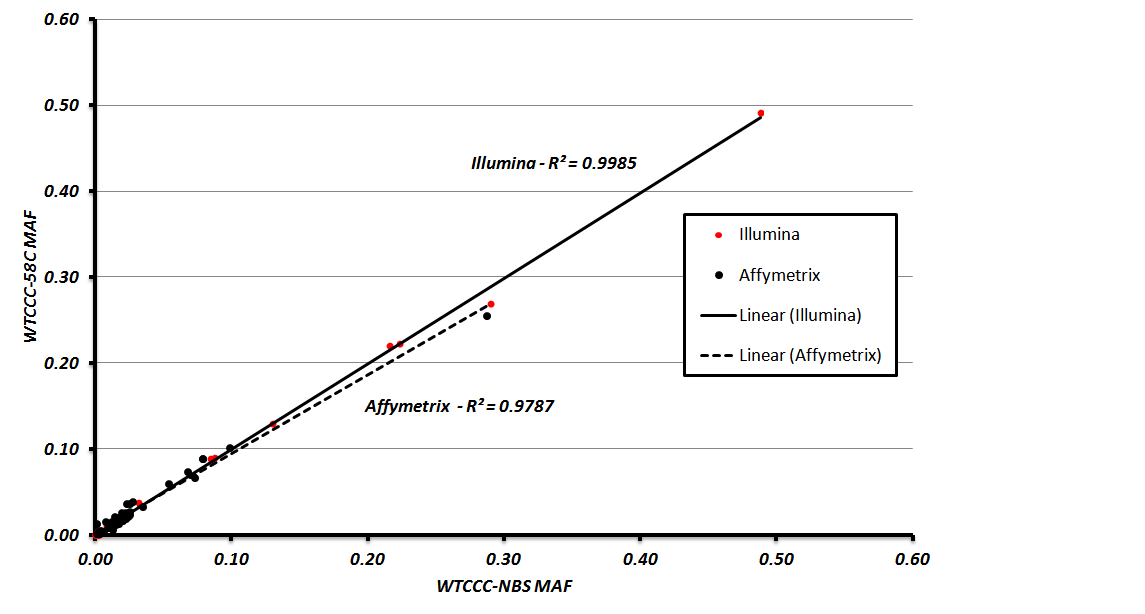

Supplement: Figure S1 — Mitochondrial DNA control allele frequencies after quality control (see methods). Comparison of the WTCCC-58C and WTCCC-NBS control cohorts. Solid line = linear regression for the Illumina data. Dotted line = linear regression for the Affymetrix data. MAF = minor allele frequency. (DOCX) [file pgen.1004369.s001.docx]

***Figure S2.***


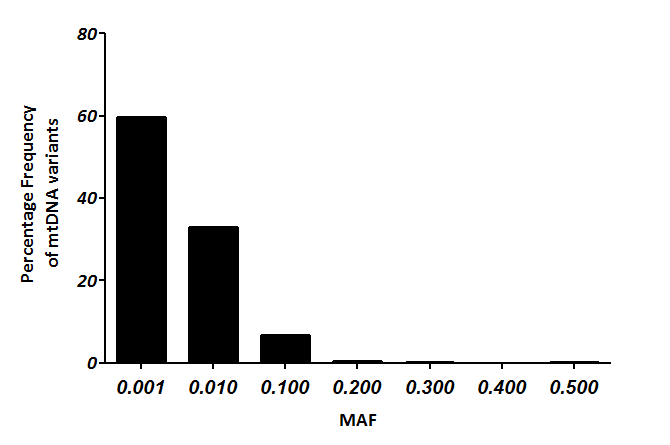


40.41% of variants with MAF>0.01

Supplement: Figure S2 — Frequency distribution histogram showing the percentage frequency of mitochondrial DNA variants in the imputation reference panel (2,873 variants from 7,729 subjects) plotted against the minor allele frequency (MAF) in the reference panel. 40.41% variants have a MAF>0.01. These variants were included in the imputation analysis. (DOCX) [file pgen.1004369.s002.docx]

***Figure S3.***


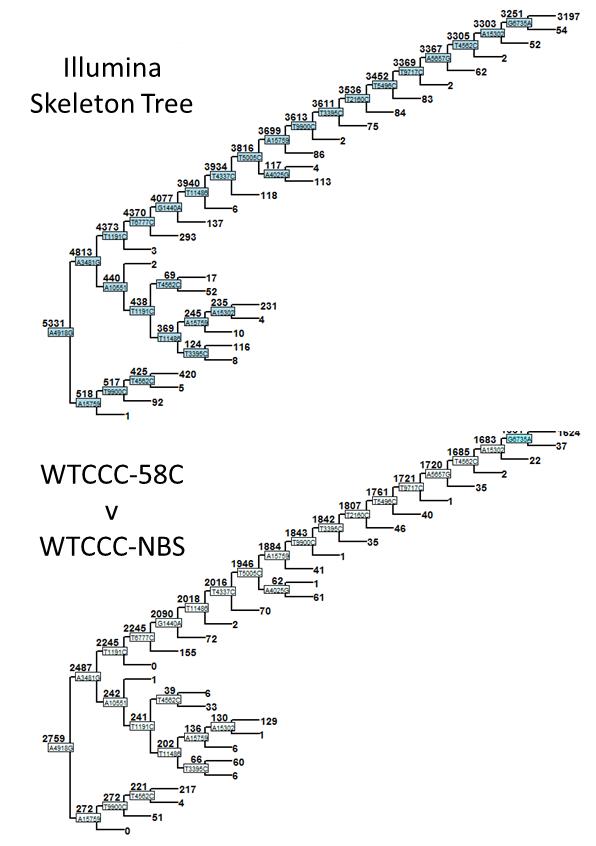


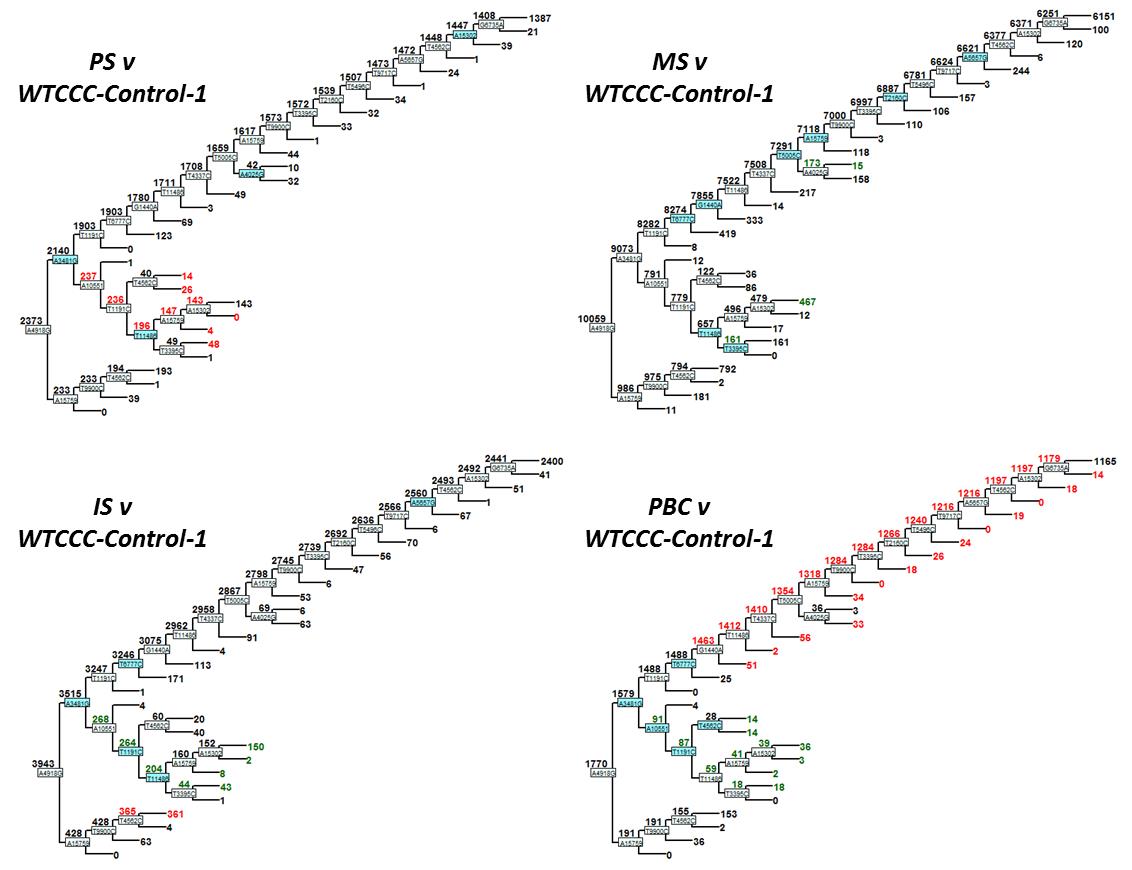


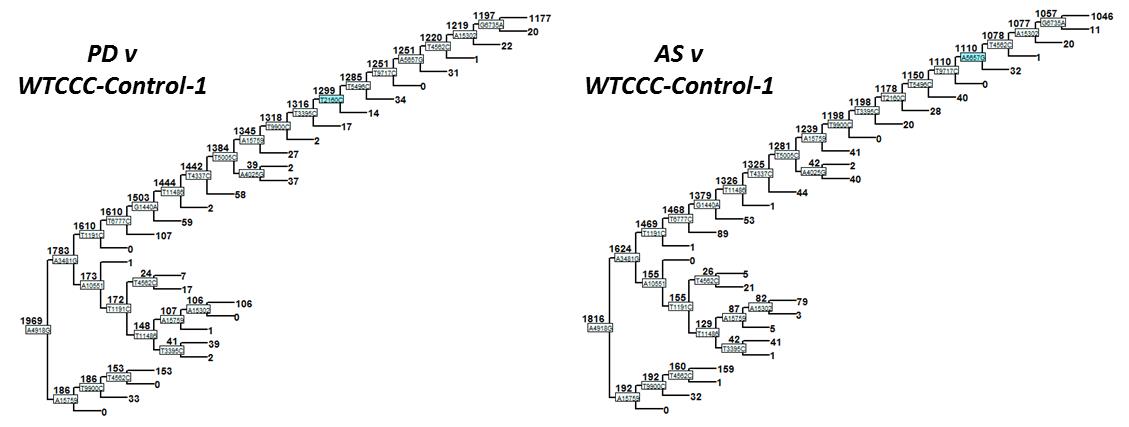


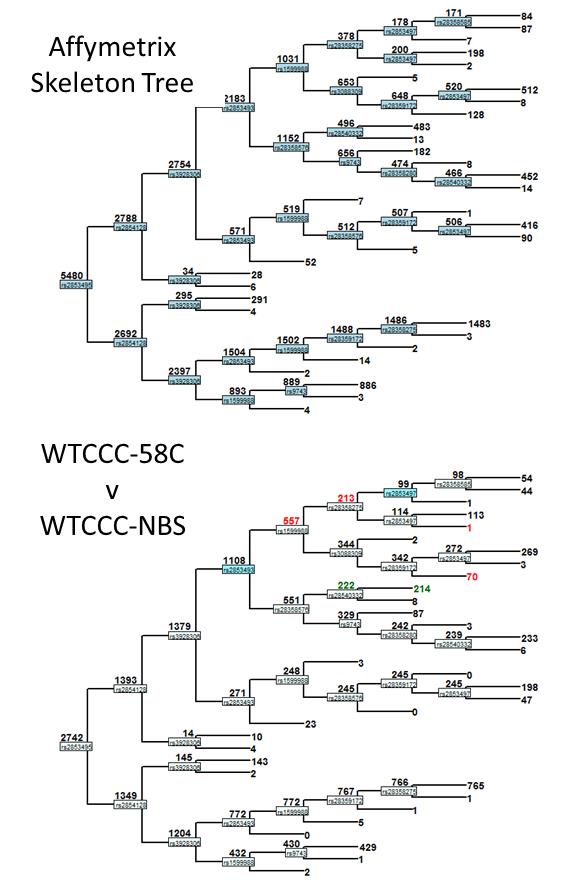


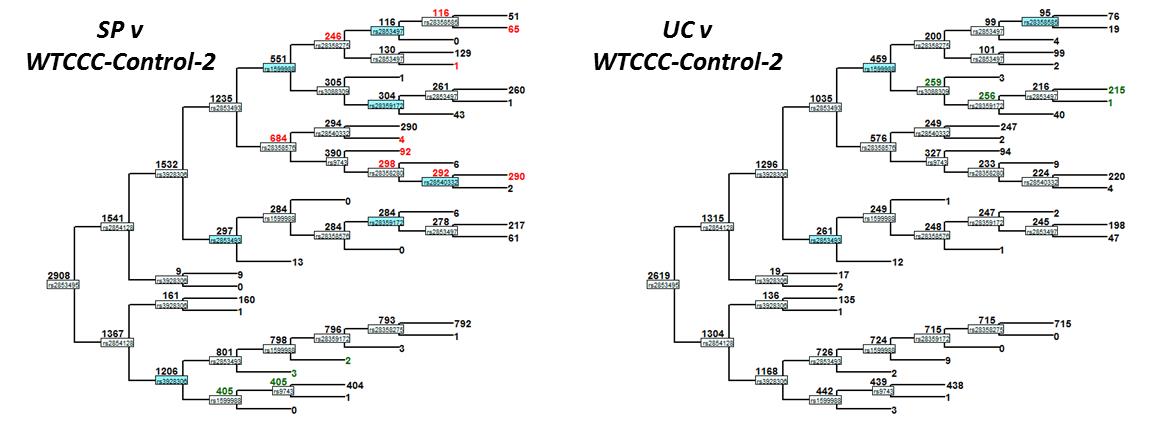


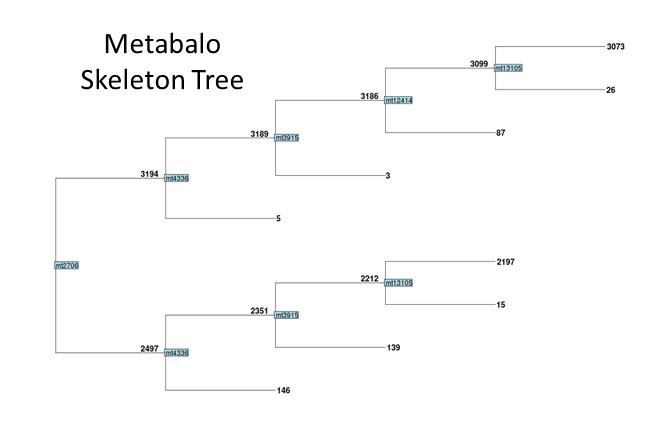


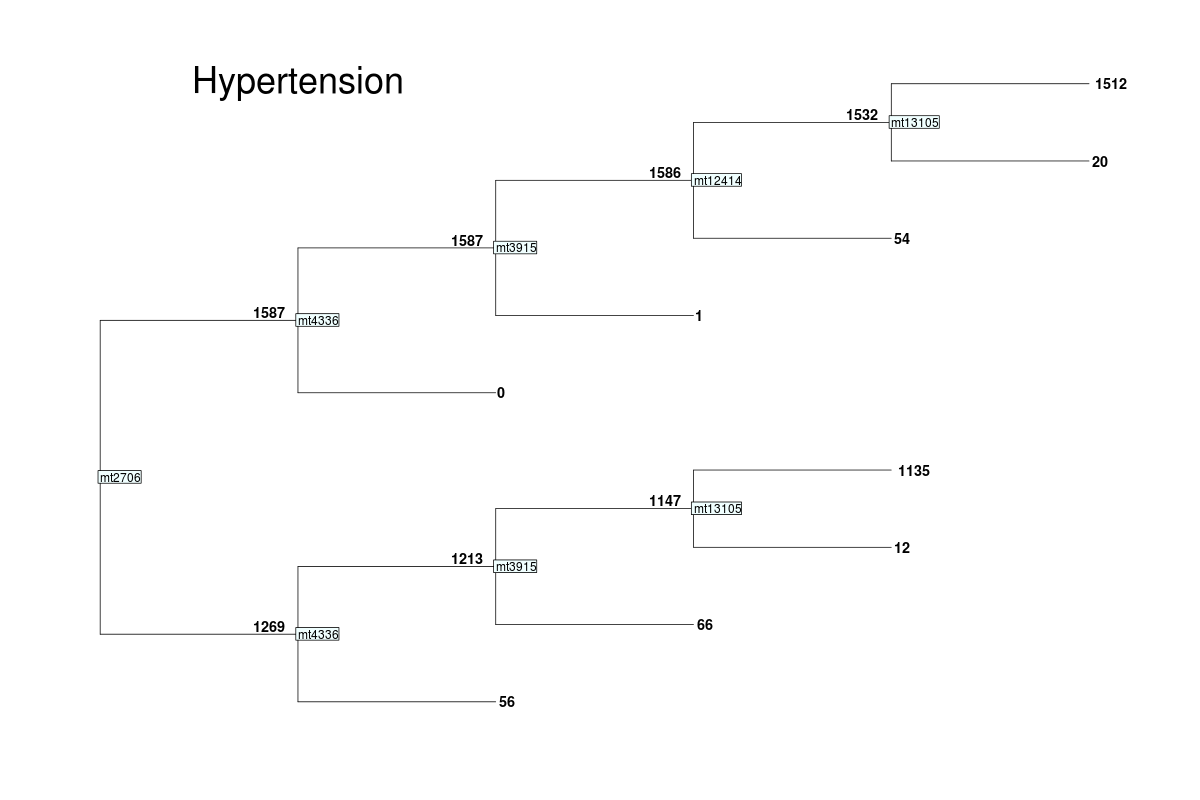


***HT v***

***WTCCC-Control-2***


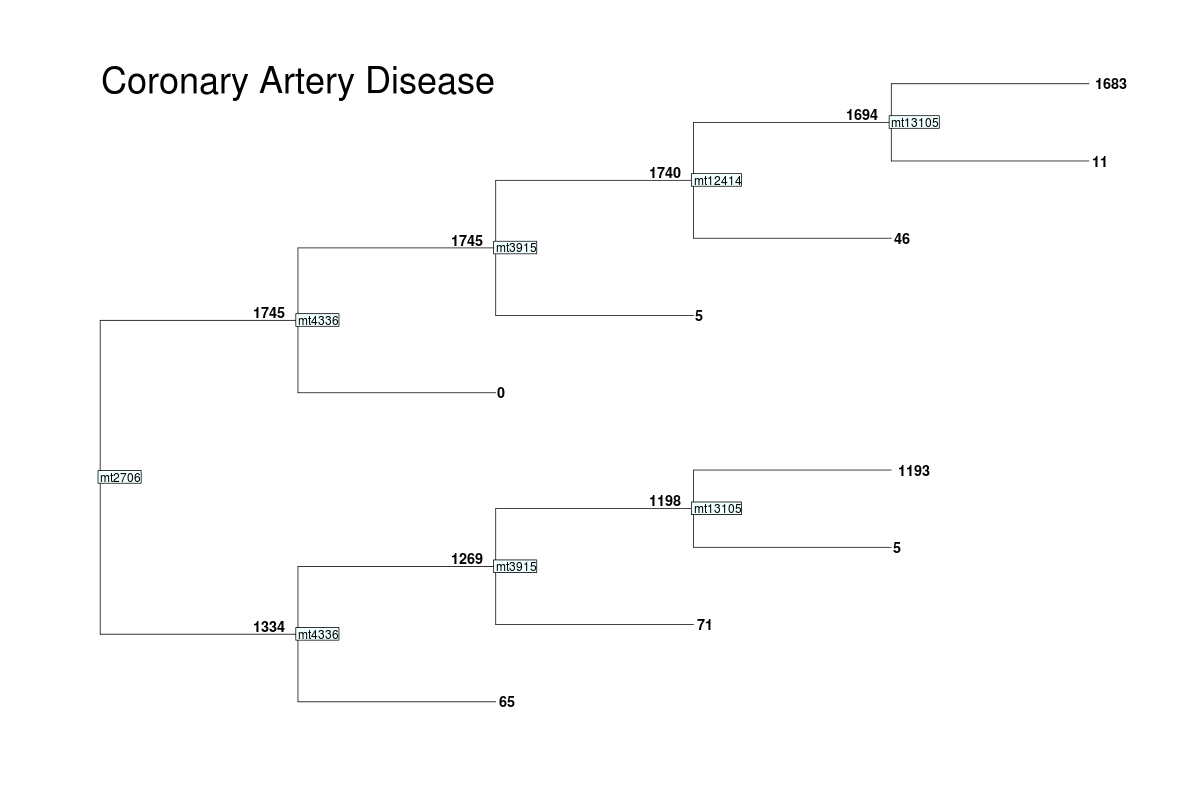


***CAD v***

***WTCCC-Control-2***


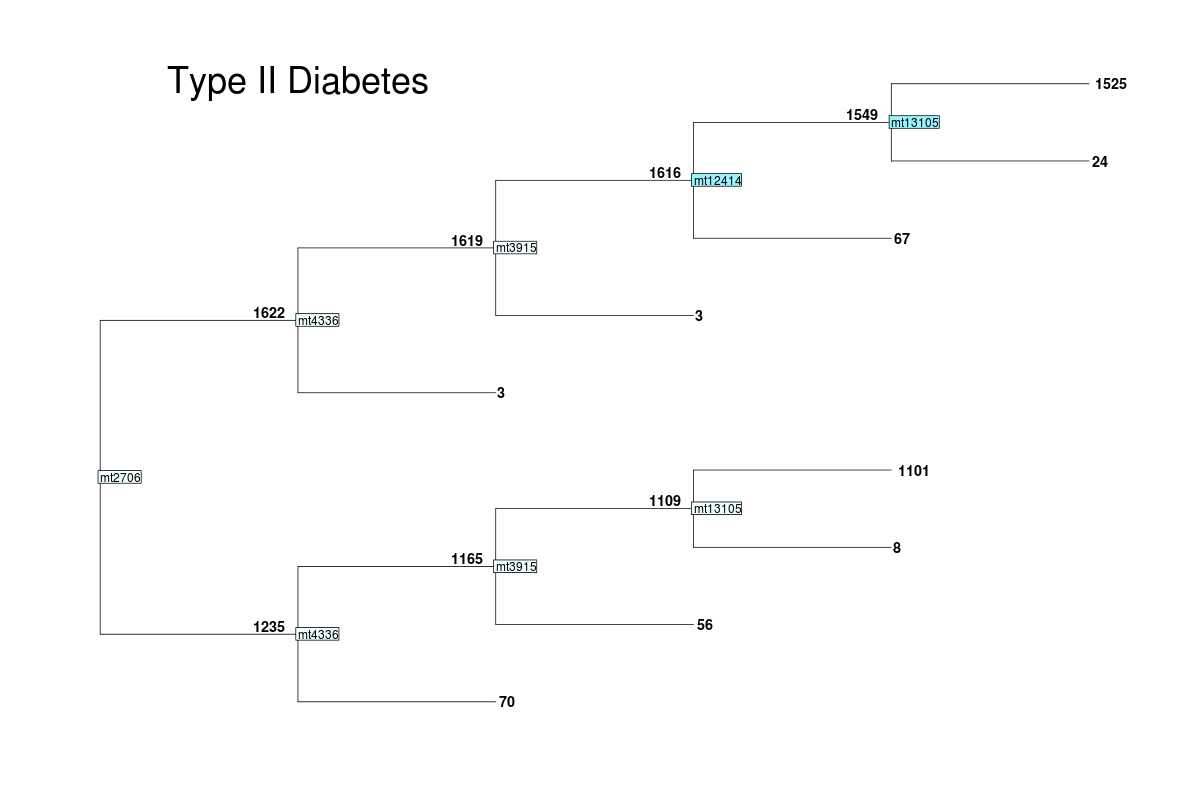


***T2D v***

***WTCCC-Control-2***

Supplement: Figure S3 — Lexical tree analysis of complex trait SNP data. Shown are skeletal tree configurations for each genotyping platform, control data comparisons (WTCCC-58C versus WTCCC-NBS for each platform, showing no significant haplotype associations) and case-control comparisons on each skeletal tree. Numbers indicate nodal frequencies and significant associations are highlighted in colour, where blue boxes indicate a protective haplotype association. AS = Ankylosing spondylitis, IS = ischaemic stroke, MS = multiple sclerosis, PD = Parkinson's disease, PBC = primary biliary cirrhosis, PS = psoriasis, SP = schizophrenia, UC = ulcerative colitis, CAD = coronary artery disease, HT = hypertension, and T2D = type 2 diabetes. (DOCX) [file pgen.1004369.s003.docx]

***Figure S4.***

**
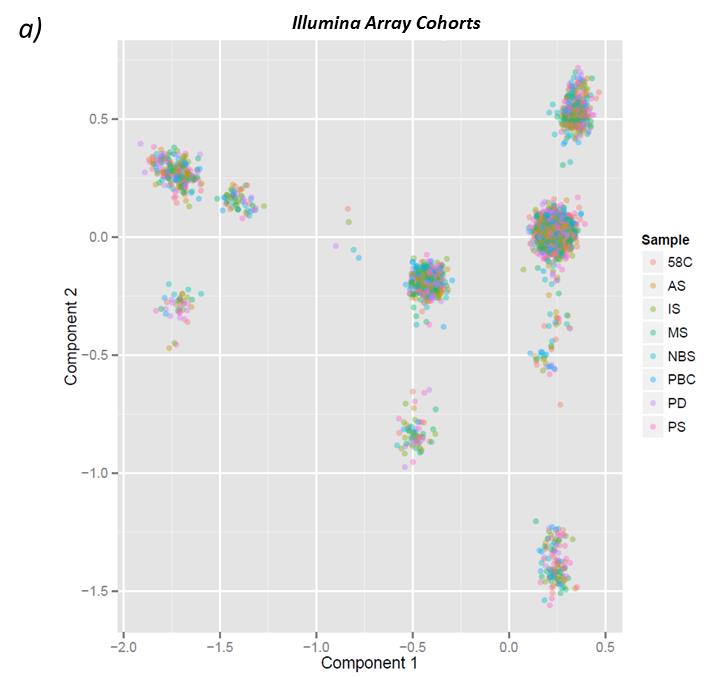
**


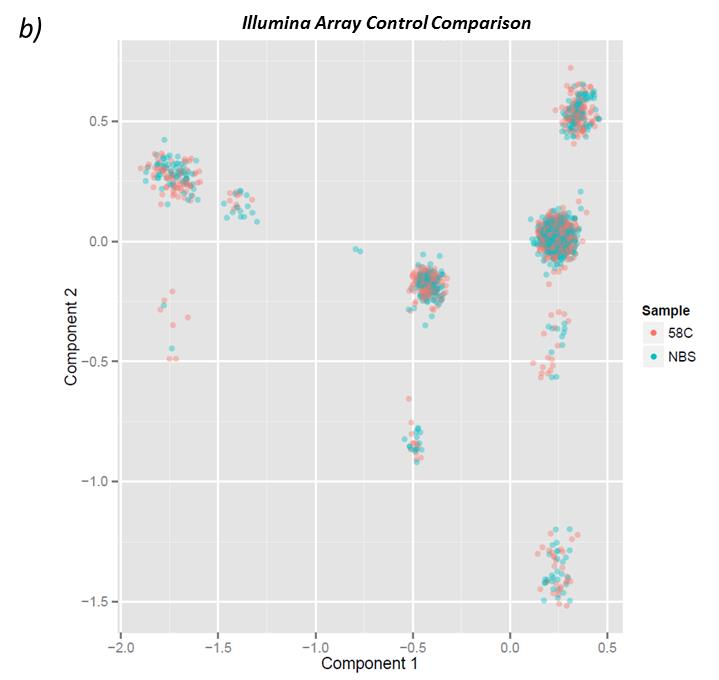


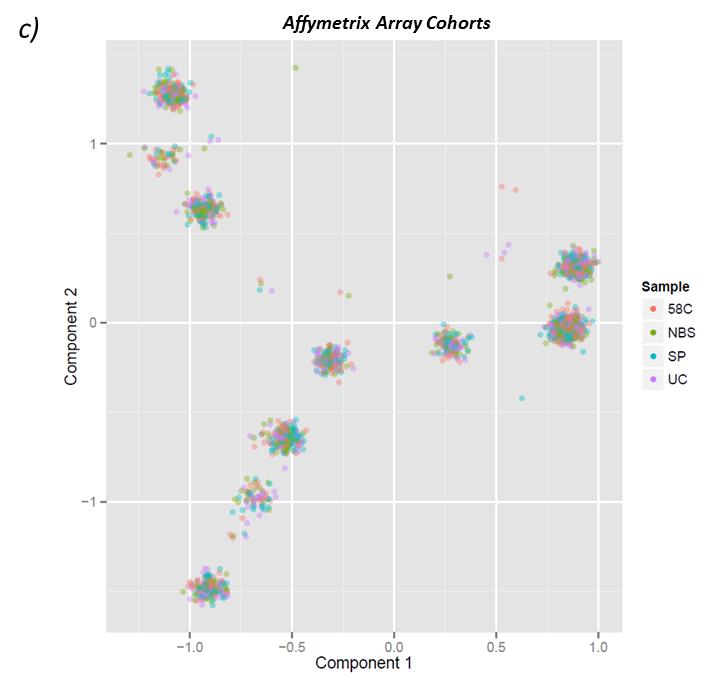


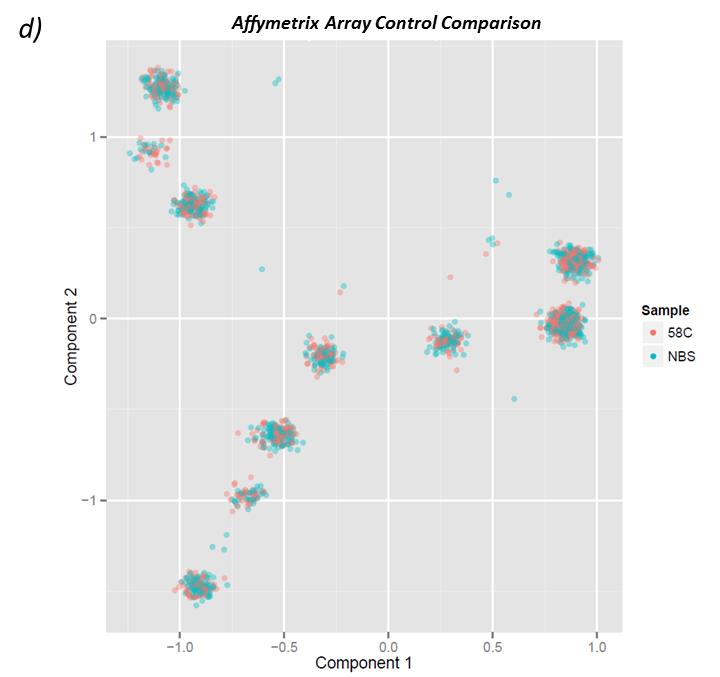


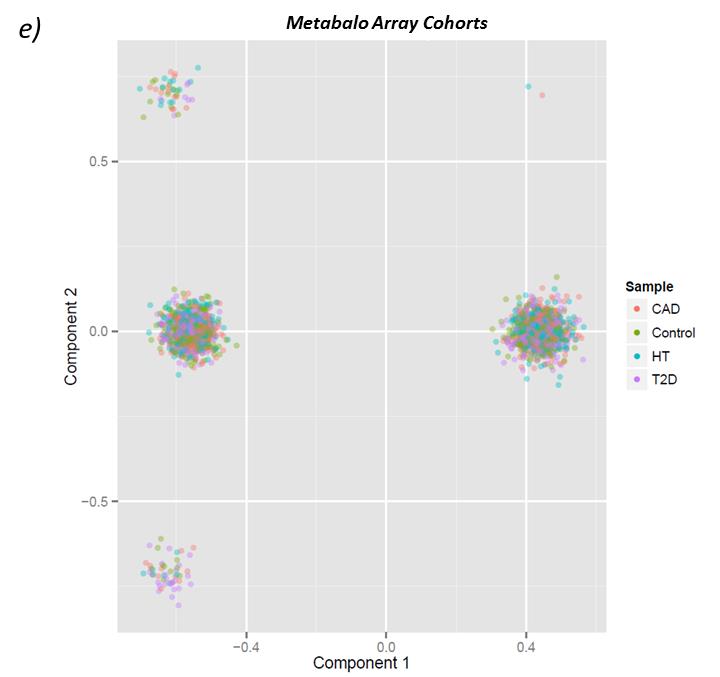

Supplement: Figure S4 — – PCA analysis of mtDNA variants, showing clustering of: a) combined Illumina array cohorts (AS, IS, MS, NBS, PBC, PD, PS, WTCCC-58C and WTCCC-NBS); b) Illumina genotype controls only (WTCCC-58C and WTCCC-NBS); c) combined Affymetrix array cohorts (SP, UC, WTCCC-58C and WTCCC-NBS); d) Affymetrix genotype controls only (WTCCC-58C and WTCCC-NBS) and e) combined Metabalo array cohorts (T2D, CAD, HT and controls [previously combined WTCCC-58C and WTCCC-NBS]). (DOCX) [file pgen.1004369.s004.docx]
