# Supplementary material for: Recent Mitochondrial DNA Mutations Increase the Risk of Developing Common Late-Onset Human Diseases
Source: PLoS Genet. 2014 May 22;10(5):e1004369. doi: 10.1371/journal.pgen.1004369 (PMC4031051; doi:10.1371/journal.pgen.1004369)
Supplement: Table S1 — Impact of quality control procedure on the number of samples and genotypes (see methods). MAF = minor allele frequency. (DOCX) [file pgen.1004369.s005.docx]

***Table S1.***

|  |  |  | ***Pre-QC*** | |  | ***Individual Removal*** | |  | ***Variant Removal*** | |  | ***Individual Removal*** | ***Final Datasets*** | |
| --- | --- | --- | --- | --- | --- | --- | --- | --- | --- | --- | --- | --- | --- | --- |
| ***Cohort*** | ***Array*** |  | ***Samples*** | ***SNPs*** |  | ***Missing Phenotypes*** | ***--mind 0.01*** |  | ***--geno 0.01*** | ***MAF >5% call rate = 95%*** |  | ***Non-European Samples*** | ***Samples*** | ***SNPs*** |
|  |  |  |  |  |  |  |  |  |  | ***MAF <5% call rate = 99%*** |  |  |  |  |
|  |  |  |  |  |  |  |  |  |  |  |  |  |  |  |
| Psoriasis | Illumina 610K |  | 2622 | 138 |  | 45 | 513 |  | 11 | 79 |  | 38 | 2026 | 48 |
| Multiple Sclerosis | Illumina 610K |  | 11376 | 138 |  | 845 | 1619 |  | 19 | 73 |  | 103 | 8809 | 42 |
| Ischemic Stroke | Illumina 610K |  | 4205 | 138 |  | 0 | 32 |  | 5 | 61 |  | 12 | 4161 | 56 |
| Primary Biliary Cirrhosis | Illumina 610K |  | 1921 | 138 |  | 0 | 0 |  | 6 | 70 |  | 0 | 1921 | 124 |
| Parkinson' Disease | Illumina 610K |  | 2197 | 138 |  | 23 | 426 |  | 9 | 79 |  | 23 | 1725 | 45 |
| Ankylosing Spondylitis | Illumina 610K |  | 2005 | 138 |  | 22 | 231 |  | 12 | 75 |  | 19 | 1733 | 44 |
| Type-2 Diabetes | MetabaloChip |  | 2975 | 135 |  | 0 | 537 |  | 65 | 58 |  | 15 | 2423 | 13 |
| Coronary Artery Disease | MetabaloChip |  | 3125 | 135 |  | 90 | 130 |  | 62 | 63 |  | 15 | 2890 | 19 |
| Hypertension | MetabaloChip |  | 2943 | 135 |  | 0 | 221 |  | 64 | 62 |  | 8 | 2714 | 13 |
| Ulcerative Colitis | Affymetrix SNP 6.0 |  | 2869 | 445 |  | 0 | 0 |  | 2 | 395 |  | 14 | 2855 | 126 |
| Schizophrenia | Affymetrix SNP 6.0 |  | 3070 | 445 |  | 0 | 12 |  | 2 | 397 |  | 0 | 3058 | 123 |
|  |  |  |  |  |  |  |  |  |  |  |  |  |  |  |
| WTCCC-58C | Illumina 1.2M |  | 2930 | 138 |  | 18 | 2894 |  | 32 | 49 |  | 18 | 2539 | 57 |
| WTCCC-NBS | Illumina 1.2M |  | 2728 | 138 |  | 25 | 2679 |  | 26 | 49 |  | 25 | 2363 | 63 |
| WTCCC-58C | Affymetrix SNP 6.0 |  | 2997 | 445 |  | 0 | 2997 |  | 52 | 324 |  | 0 | 2437 | 69 |
| WTCCC-NBS | Affymetrix SNP 6.0 |  | 2987 | 445 |  | 0 | 2897 |  | 35 | 310 |  | 0 | 2805 | 100 |
| WTCCC-58C+NBS | MetabaloChip |  | 5841 | 135 |  | 0 | 5841 |  | 72 | 45 |  | 0 | 5367 | 18 |
|  |  |  |  |  |  |  |  |  |  |  |  |  |  |  |
