# Supplementary material for: Recent Mitochondrial DNA Mutations Increase the Risk of Developing Common Late-Onset Human Diseases
Source: PLoS Genet. 2014 May 22;10(5):e1004369. doi: 10.1371/journal.pgen.1004369 (PMC4031051; doi:10.1371/journal.pgen.1004369)
Supplement: Table S2 — Impact of quality control procedure when combining control data (see methods). (DOCX) [file pgen.1004369.s006.docx]

***Table S2.***

|  |  | ***Pre QC*** | | ***MAF 0.00001*** | ***--geno 0.01*** | ***--mind 0.01*** | ***--assoc*** |  | ***Final Datasets*** |  |  |
| --- | --- | --- | --- | --- | --- | --- | --- | --- | --- | --- | --- |
| ***Controls merged*** | ***Array*** | ***N=*** | ***SNPs*** | ***SNPs remaining*** | ***SNPs remaining*** | ***Samples remaining*** | ***SNPs remaining*** |  | ***Control cohort*** | ***N=*** | ***SNPs*** |
|  |  |  |  |  |  |  |  |  |  |  |  |
| 58C+NBS | Illumina 1.2M | 4902 | 74 | 73 | 46 | 4902 | 46 |  | ***WTCCC-Control-1*** | ***4902*** | ***46*** |
| 58C+NBS | Affymetrix SNP 6.0 | 5242 | 128 | 114 | 45 | 5033 | 45 |  | ***WTCCC-Control-2*** | ***5033*** | ***45*** |
| 58C+NBS | MetabaloChip | 5367 | 18 | 18 | 18 | 5367 | 18 |  | ***WTCCC-Control-3*** | ***5367*** | ***18*** |
