# Supplementary material for: Recent Mitochondrial DNA Mutations Increase the Risk of Developing Common Late-Onset Human Diseases
Source: PLoS Genet. 2014 May 22;10(5):e1004369. doi: 10.1371/journal.pgen.1004369 (PMC4031051; doi:10.1371/journal.pgen.1004369)
Supplement: Table S3 — Association between imputed mitochondrial DNA variants and eight complex diseases, showing the corresponding control cohort, array SNP ID, variant position in the mitochondrial genome (rCRS, NC_012920), minor allele frequency in cases and controls (A1-cases and A1-Cont. respectively), case-control comparison (chi-square test P, na = not available in primary analysis), imputed significance (P) and odds ratio (OR). Hap = corresponding major and sub mitochondrial haplogroup. (DOCX) [file pgen.1004369.s007.docx]

***Table S3.***

|  |  |  |  |  |  | ***Case Control*** | | ***Imputation*** | |  |  |
| --- | --- | --- | --- | --- | --- | --- | --- | --- | --- | --- | --- |
| ***Case Cohort*** | ***Control Comparison*** | ***SNP Array ID*** | ***rCRS*** | ***A1 Cases*** | ***A1 Cont.*** | ***P*** | ***OR (95% CI)*** | ***P*** | ***OR*** | ***Major mtDNA Hap*** | ***Minor mtDNA Hap*** |
|  |  |  |  |  |  |  |  |  |  |  |  |
| Psoriasis | WTCCC-Control-1 | MitoA15302G | m.15301 | 0.020 | 0.012 | 6.55E-03 | 1.71 (1.15-2.53) | 1.14E-02 | 1.71 | Homoplastic(U/K) | U5a2b4/K1b2b |
|  |  | MitoC310T | m.310 | 0.260 | 0.217 | *na* | *na* | 1.14E-02 | 1.71 | U | U4a2 |
|  |  | MitoA9668G | m.9667 | 0.020 | 0.010 | *na* | *na* | 1.18E-02 | 1.88 | Homoplastic(J/U) | J1b2a/ U5a1b |
|  |  |  |  |  |  |  |  |  |  |  |  |
| Multiple Sclerosis | WTCCC-Control-1 | MitoA5657G | m.5656 | 0.024 | 0.012 | 1.96E-07 | 2.07 (1.56-2.74) | 1.92E-04 | 2.03 | U | U5b1 |
|  |  | MitoG6261A | m.6260 | 0.015 | 0.024 | 1.44E-04 | 0.63 (0.49-0.80) | 2.78E-03 | 0.63 | Homoplastic(H/K) | H2a1d/K1a4a |
|  |  | MitoT6777C | m.6776 | 0.042 | 0.054 | 1.91E-03 | 0.78 (0.66-0.91) | 2.12E-02 | 0.78 | H | H3 |
|  |  | MitoT10035C | m.10034 | 0.026 | 0.033 | 1.20E-02 | 0.78 (0.63-0.95) | 4.25E-02 | 0.77 | N1(I) | I |
|  |  | MitoG10399A | m.10398 | 0.207 | 0.230 | 9.62E-04 | 0.87 (0.80-0.95) | 1.54E-02 | 0.87 | Homoplastic(J/K) | J/K1 |
|  |  | MitoT11486C | m.11485 | 0.017 | 0.026 | 7.19E-04 | 0.67 (0.53-0.85) | 8.26E-03 | 0.67 | K | K1a4 |
|  |  | MitoC150T | m.150 | 0.112 | 0.093 | *na* | *na* | 1.79E-04 | 1.24 | Homoplastic | - |
|  |  | MitoC310T | m.310 | 0.250 | 0.217 | *na* | *na* | 1.62E-04 | 1.20 | U | U4a2 |
|  |  | MitoC499T | m.497 | 0.051 | 0.062 | *na* | *na* | 3.59E-02 | 0.82 | K | K1a |
|  |  | MitoT3198C | m.3197 | 0.097 | 0.075 | *na* | *na* | 7.70E-04 | 1.32 | U | U5 |
|  |  | MitoG8270A | m.8269 | 0.026 | 0.036 | *na* | *na* | 1.14E-02 | 0.72 | Homoplastic | - |
|  |  | MitoG9478A | m.9477 | 0.095 | 0.079 | *na* | *na* | 1.39E-02 | 1.22 | U | U5 |
|  |  | MitoC11841T | m.11840 | 0.012 | 0.021 | *na* | *na* | 1.18E-03 | 0.58 | K | K1a4a1 |
|  |  | MitoT13741C | m.13740 | 0.012 | 0.021 | *na* | *na* | 1.25E-03 | 0.58 | Homoplastic(H/K) | H1/K1a4a |
|  |  | MitoT14799C | m.14798 | 0.133 | 0.150 | *na* | *na* | 2.05E-02 | 0.87 | K | - |
|  |  | MitoG16393A | m.16391 | 0.025 | 0.032 | *na* | *na* | 3.90E-02 | 0.77 | N1(I) | N1a1b |
|  |  |  |  |  |  |  |  |  |  |  |  |
| Ischemic Stroke | WTCCC-Control-1 | MitoT1191C | m.1189 | 0.058 | 0.076 | 4.52E-04 | 0.75 (0.63-0.88) | 1.56E-02 | 0.76 | K | K1 |
|  |  | MitoG6261A | m.6260 | 0.013 | 0.024 | 5.30E-05 | 0.52 (0.38-0.72) | 1.33E-03 | 0.53 | Homoplastic(H/K) | H2a1d/K1a4a |
|  |  | MitoG10399A | m.10398 | 0.209 | 0.230 | 1.07E-02 | 0.88 (0.80-0.97) | 4.90E-02 | 0.88 | Homoplastic(J/K) | J/K1 |
|  |  | MitoA10551G | m.10550 | 0.074 | 0.089 | 7.56E-03 | 0.82 (0.71-0.95) | 4.67E-02 | 0.82 | K | - |
|  |  | MitoT11486C | m.11485 | 0.014 | 0.026 | 4.89E-05 | 0.53 (0.39-0.72) | 3.62E-03 | 0.57 | K | K1a4 |
|  |  | MitoC310T | m.310 | 0.239 | 0.217 | *na* | *na* | 3.94E-02 | 1.13 | U | U4a2 |
|  |  | MitoC499T | m.497 | 0.045 | 0.062 | *na* | *na* | 5.03E-03 | 0.72 | K | K1a |
|  |  | MitoT3198C | m.3197 | 0.089 | 0.070 | *na* | *na* | 1.01E-02 | 1.30 | U | U5 |
|  |  | MitoT11300C | m.11299 | 0.074 | 0.089 | *na* | *na* | 4.64E-02 | 0.81 | K | - |
|  |  | MitoC11841T | m.11840 | 0.011 | 0.021 | *na* | *na* | 8.78E-04 | 0.49 | K | K1a4a1 |
|  |  | MitoT13741C | m.13740 | 0.011 | 0.021 | *na* | *na* | 9.47E-04 | 0.49 | Homoplastic(H/K) | H1/K1a4a |
|  |  | MitoC14168T | m.14167 | 0.074 | 0.089 | *na* | *na* | 4.56E-02 | 0.81 | K | U8b(K) |
|  |  | MitoC16298T | m.16296 | 0.060 | 0.049 | *na* | *na* | 3.66E-02 | 1.24 | T | T2 |
|  |  |  |  |  |  |  |  |  |  |  |  |
| Primary Biliary Cirrhosis | WTCCC-Control-1 | MitoT1191C | m.1189 | 0.036 | 0.076 | 2.80E-07 | 0.46 (0.36-0.60) | 3.77E-02 | 0.74 | K | K1 |
|  |  | MitoT6777C | m.6776 | 0.013 | 0.054 | 2.06E-08 | 0.24 (0.16-0.36) | 3.69E-07 | 0.30 | H | H3 |
|  |  | MitoC499T | m.497 | 0.037 | 0.062 | *na* | *na* | 1.28E-03 | 0.58 | K | K1a |
|  |  | MitoT16173C | m.16172 | 0.111 | 0.059 | *na* | *na* | 1.44E-12 | 1.99 | Homoplastic | - |
|  |  | MitoC16280T | m.16278 | 0.070 | 0.040 | *na* | *na* | 6.26E-06 | 1.80 | Homoplastic | - |
|  |  | MitoC16294T | m.16292 | 0.036 | 0.017 | *na* | *na* | 1.64E-04 | 2.16 | Homoplastic | - |
|  |  | MitoC16296T | m.16294 | 0.177 | 0.107 | *na* | *na* | 4.51E-11 | 1.80 | Homoplastic | - |
|  |  |  |  |  |  |  |  |  |  |  |  |
| Parkinson' Disease | WTCCC-Control-1 | MitoT2160C | m.2158 | 0.010 | 0.015 | 3.21E-02 | 0.54 (0.31-0.95) | 3.21E-02 | 0.54 | J | J1b1a |
|  |  | MitoT3198C | m.3197 | 0.099 | 0.047 | 9.99E-04 | 2.24 (1.83-2.75) | 1.69E-02 | 1.36 | U | U5 |
|  |  | MitoG10399A | m.10398 | 0.199 | 0.230 | 6.67E-03 | 0.83 (0.73-0.95) | 4.43E-02 | 0.84 | Homoplastic(J/K) | J/K1 |
|  |  | MitoC310T | m.310 | 0.257 | 0.217 | *na* | *na* | 5.40E-03 | 1.25 | U | U4a2 |
|  |  | MitoG9478A | m.9477 | 0.098 | 0.078 | *na* | *na* | 4.70E-02 | 1.28 | U | U5 |
|  |  | MitoA12613G | m.12612 | 0.091 | 0.113 | *na* | *na* | 4.11E-02 | 0.79 | J | - |
|  |  | MitoC16070T | m.16069 | 0.091 | 0.112 | *na* | *na* | 4.13E-02 | 0.79 | J | - |
|  |  |  |  |  |  |  |  |  |  |  |  |
| Ankylosing Spondylitis | WTCCC-Control-1 | MitoA9668G | m.9667 | 0.018 | 0.010 | 1.08E-02 | 1.76 (1.13-2.73) | 4.63E-02 | 1.73 | Homoplastic(J/U) | J1b2a/ U5a1b |
|  |  | MitoG10399A | m.10398 | 0.195 | 0.230 | 2.26E-03 | 0.81 (0.71-0.93) | 3.71E-02 | 0.83 | Homoplastic(J/K) | J/K1 |
|  |  | MitoC64T | m.64 | 0.041 | 0.013 | *na* | *na* | 1.72E-07 | 3.32 | Homoplastic | - |
|  |  | MitoT146C | m.146 | 0.182 | 0.150 | *na* | *na* | 7.03E-03 | 1.26 | Homoplastic | - |
|  |  | MitoC150T | m.150 | 0.112 | 0.092 | *na* | *na* | 2.58E-02 | 1.25 | Homoplastic | - |
|  |  | MitoA153G | m.153 | 0.058 | 0.013 | *na* | *na* | 2.38E-10 | 4.57 | Homoplastic | - |
|  |  | MitoC310T | m.310 | 0.265 | 0.217 | *na* | *na* | 8.88E-04 | 1.30 | U | U4a2 |
|  |  | MitoT1702C | m.1700 | 0.024 | 0.015 | *na* | *na* | 3.89E-02 | 1.60 | Homoplastic(U) | U5a1a/U2d |
|  |  | MitoT3198C | m.3197 | 0.110 | 0.075 | *na* | *na* | 4.36E-04 | 1.53 | U | U5 |
|  |  | MitoG9478A | m.9477 | 0.110 | 0.078 | *na* | *na* | 1.85E-03 | 1.45 | U | U5 |
|  |  | MitoT13618C | m.13617 | 0.109 | 0.088 | *na* | *na* | 4.46E-02 | 1.26 | U | U5 |
|  |  | MitoA14794G | m.14793 | 0.065 | 0.049 | *na* | *na* | 2.84E-02 | 1.34 | U | U5a |
|  |  | MitoA15219G | m.15218 | 0.049 | 0.034 | *na* | *na* | 1.62E-02 | 1.48 | U | U5a1 |
|  |  |  |  |  |  |  |  |  |  |  |  |
| Ulcerative Colitis | WTCCC-Control-2 | rs2001030 | m.1438 | 0.028 | 0.013 | 9.63E-07 | 2.17 (1.58-2.99) | 2.90E-03 | 2.08 | H | H2 |
|  |  | rs28754574 | m.10135 | 0.017 | 0.009 | 3.51E-04 | 2.03 (1.37-3.01) | 2.78E-04 | 2.01 | - | - |
|  |  | rs2853496 | m.11914 | 0.025 | 0.008 | 9.35E-10 | 2.99 (2.07-4.32) | 7.70E-04 | 2.71 | Homoplastic | - |
|  |  | rs2853511 | m.16093 | 0.058 | 0.033 | 1.16E-07 | 1.78 (1.44-2.21) | 7.76E-04 | 1.67 | Homoplastic | - |
|  |  | rs2857289 | m.16257 | 0.063 | 0.022 | 4.29E-22 | 2.97 (2.36-3.74) | 2.94E-10 | 2.80 | Homoplastic | - |
|  |  | MitoG711A | m.709 | 0.154 | 0.173 | *na* | *na* | 4.02E-02 | 0.87 | Homoplastic | - |
|  |  | MitoG1890A | m.1888 | 0.027 | 0.075 | *na* | *na* | 7.69E-10 | 0.34 | T | - |
|  |  | rs2854131 | m.3197 | 0.092 | 0.071 | *na* | *na* | 9.25E-03 | 1.33 | U | U5 |
|  |  | MitoG4770A | m.4769 | 0.023 | 0.012 | *na* | *na* | 7.78E-03 | 2.03 | H | H2a |
|  |  | MitoA4918G | m.4917 | 0.031 | 0.078 | *na* | *na* | 2.46E-09 | 0.38 | T | - |
|  |  | MitoG8698A | m.8697 | 0.027 | 0.075 | *na* | *na* | 6.58E-10 | 0.34 | T | - |
|  |  | MitoG9478A | m.9477 | 0.082 | 0.057 | *na* | *na* | 2.48E-04 | 1.48 | U | U5 |
|  |  | MitoG13369A | m.13368 | 0.026 | 0.075 | *na* | *na* | 6.05E-10 | 0.33 | T | - |
|  |  | rs2853503 | m.13617 | 0.091 | 0.070 | *na* | *na* | 1.11E-02 | 1.32 | U | U5 |
|  |  | rs28357684 | m.15043 | 0.052 | 0.037 | *na* | *na* | 7.60E-03 | 1.44 | N1(I) | N1a1 |
|  |  | rs2853506 | m.15218 | 0.039 | 0.015 | *na* | *na* | 2.89E-06 | 2.60 | U | U5a1 |
|  |  | rs2853510 | m.15924 | 0.069 | 0.045 | *na* | *na* | 2.14E-04 | 1.58 | Homoplastic | - |
|  |  | MitoG15929A | m.15928 | 0.029 | 0.076 | *na* | *na* | 1.83E-09 | 0.37 | T | - |
|  |  | MitoC16193T | m.16192 | 0.061 | 0.045 | *na* | *na* | 3.45E-03 | 1.39 | Homoplastic | - |
|  |  | rs2857290 | m.16270 | 0.080 | 0.056 | *na* | *na* | 2.62E-04 | 1.48 | U | U5 |
|  |  | MitoC16296T | m.16294 | 0.039 | 0.087 | *na* | *na* | 6.11E-09 | 0.43 | Homoplastic | - |
|  |  | MitoT16306C | m.16304 | 0.059 | 0.080 | *na* | *na* | 2.00E-03 | 0.71 | Homoplastic | - |
|  |  | rs34799580 | m.16311 | 0.157 | 0.125 | *na* | *na* | 4.61E-03 | 1.30 | Homoplastic | - |
|  |  |  |  |  |  |  |  |  |  |  |  |
| Schizophrenia | WTCCC-Control-2 | rs3928306 | m.3010 | 0.239 | 0.260 | 2.79E-02 | 0.89 (0.81-0.99) | 2.52E-03 | 0.81 | Homoplastic(H/J) | H1/J1 |
|  |  | rs28358286 | m.11674 | 0.023 | 0.015 | 6.17E-03 | 1.55 (1.13-2.13) | 1.00E-02 | 1.93 | N2(W) | - |
|  |  | rs3021089 | m.8251 | 0.073 | 0.054 | *na* | *na* | 5.23E-03 | 1.38 | Homoplastic | - |
|  |  | rs2853510 | m.15924 | 0.071 | 0.046 | *na* | *na* | 9.99E-05 | 1.58 | Homoplastic | - |
|  |  | rs2853511 | m.16093 | 0.058 | 0.035 | *na* | *na* | 4.11E-04 | 1.69 | Homoplastic | - |
|  |  | rs2853817 | m.16172 | 0.084 | 0.037 | *na* | *na* | 1.45E-10 | 2.38 | Homoplastic | - |
|  |  | rs2857289 | m.16256 | 0.069 | 0.025 | *na* | *na* | 2.69E-12 | 2.88 | Homoplastic | - |
|  |  | rs34799580 | m.16311 | 0.158 | 0.122 | *na* | *na* | 1.12E-03 | 1.34 | Homoplastic | - |
|  |  | rs28358285 | m.11299 | 0.106 | 0.084 | *na* | *na* | 9.62E-03 | 1.29 | K | - |
|  |  | rs28397767 | m.12501 | 0.050 | 0.034 | *na* | *na* | 3.78E-03 | 1.50 | N1(I) | - |
|  |  | rs28357684 | m.15043 | 0.051 | 0.037 | *na* | *na* | 1.07E-02 | 1.41 | N1(I) | N1a1 |
|  |  | rs2854122 | m.12705 | 0.087 | 0.041 | *na* | *na* | 3.47E-07 | 2.21 | Non-R (=A) | - |
|  |  | rs2854131 | m.3197 | 0.102 | 0.072 | *na* | *na* | 2.33E-04 | 1.46 | U | U5 |
|  |  | rs2853503 | m.13617 | 0.101 | 0.069 | *na* | *na* | 8.58E-05 | 1.51 | U | U5 |
|  |  | rs2853504 | m.14793 | 0.064 | 0.050 | *na* | *na* | 3.79E-02 | 1.28 | U | U5a |
|  |  | rs2853506 | m.15218 | 0.042 | 0.015 | *na* | *na* | 4.99E-08 | 2.87 | U | U5a1 |
|  |  | rs2853514 | m.16230 | 0.022 | 0.010 | *na* | *na* | 1.20E-05 | 2.21 | U | U2a |
|  |  |  |  |  |  |  |  |  |  |  |  |
