# Supplementary material for: Recent Mitochondrial DNA Mutations Increase the Risk of Developing Common Late-Onset Human Diseases
Source: PLoS Genet. 2014 May 22;10(5):e1004369. doi: 10.1371/journal.pgen.1004369 (PMC4031051; doi:10.1371/journal.pgen.1004369)
Supplement: Table S4 — Mitochondrial DNA variants used to the lexical tree analysis (see methods). Variant position in the mitochondrial genome is based on the revised Cambridge reference sequence (rCRS, NC_012920). (DOCX) [file pgen.1004369.s008.docx]

***Table S4.***

| ***Illumina 610K*** | | | ***Affymetrix SNP 6.0*** | | | ***MetabaloChip*** | | |
| --- | --- | --- | --- | --- | --- | --- | --- | --- |
| SNP ID | MAF | rCRS  Position | SNP ID | MAF | rCRS  Position | SNP ID | MAF | rCRS  Position |
| MitoA12309G | 0.218 | 12308 | rs2853495 | 0.491 | 11719 | mt2706 | 0.439 | 2706 |
| MitoA4918G | 0.096 | 4917 | rs2854128 | 0.444 | 2706 | mt4336 | 0.027 | 4336 |
| MitoA3481G | 0.088 | 3480 | rs3928306 | 0.279 | 3010 | mt3915 | 0.025 | 3915 |
| MitoA10551G | 0.088 | 10550 | rs1599988 | 0.228 | 4216 | mt12414 | 0.015 | 12414 |
| MitoT1191C | 0.076 | 1189 | rs3088309 | 0.223 | 15452 | mt13105 | 0.007 | 13105 |
| MitoA15925G | 0.058 | 15924 | rs2853493 | 0.202 | 11467 |  |  |  |
| MitoT6777C | 0.055 | 6776 | rs28359172 | 0.126 | 12612 |  |  |  |
| MitoT10239C | 0.035 | 10238 | rs28358576 | 0.118 | 1811 |  |  |  |
| MitoT10035C | 0.032 | 10034 | rs9743 | 0.075 | 9698 |  |  |  |
| MitoG1440A | 0.027 | 1438 | rs28358280 | 0.073 | 10550 |  |  |  |
| MitoT11486C | 0.025 | 11485 | rs28358275 | 0.039 | 10238 |  |  |  |
| MitoT4337C | 0.022 | 4336 | rs2857289 | 0.022 | 12007 |  |  |  |
| MitoT5005C | 0.022 | 5004 | rs2853497 | 0.021 | 3505 |  |  |  |
| MitoA4025G | 0.021 | 4024 | rs28358585 | 0.018 | 11947 |  |  |  |
| MitoA15759G | 0.019 | 15758 | rs28359168 | 0.017 | 14365 |  |  |  |
| MitoT9900C | 0.017 | 9989 |  |  |  |  |  |  |
| MitoT3395C | 0.016 | 3394 |  |  |  |  |  |  |
| MitoT2160C | 0.016 | 2158 |  |  |  |  |  |  |
| MitoT5496C | 0.016 | 5495 |  |  |  |  |  |  |
| MitoT9717C | 0.013 | 9716 |  |  |  |  |  |  |
| MitoA5657G | 0.012 | 5656 |  |  |  |  |  |  |
| MitoA15302G | 0.011 | 15301 |  |  |  |  |  |  |
| MitoT4562C | 0.011 | 4561 |  |  |  |  |  |  |
| MitoG6735A | 0.010 | 6734 |  |  |  |  |  |  |
